# The effect of schistosomiasis and soil-transmitted helminths on expressive language skills among African preschool children

**DOI:** 10.1186/s12879-022-07260-2

**Published:** 2022-03-18

**Authors:** Xolisile I. Mazibuko, Moses Chimbari

**Affiliations:** Present Address: School of Nursing and Public Health, College of Health Sciences, University of KwaZulu-Natal UKZN, George Campbell Building, Howard College Campus, 4001 Durban, South Africa

**Keywords:** Schistosomiasis, Soil-transmitted helminths, Expressive language, Preschool, isiZulu, Africa

## Abstract

**Background:**

Schistosomiasis and soil transmitted helminths (STH) have been associated with compromised child development. We determined the effect of schistosomiasis and STH on expressive language skills among isiZulu speaking preschool children focusing on the variables: age, gender, school and stunting.

**Methods:**

We quantitatively compared the performance of a cohort of infected and non-infected children using a 2 phased approach. In phase 1 infected children were treated with praziquantel and matched with non-infected children and both groups were tested for expressive language performance. In phase 2 both groups of children were re-tested for expressive language skills using a similar but modified test. The participants were 106 preschool children between the age of 4 and 6 years, 11 months. The Developmental Language Test was adapted as a linguistically and culturally appropriate tool for assessing isiZulu expressive language skills.

**Results:**

The overall performance of the children in phases 1 and 2 were statistically similar. There was significant Pearson’s correlation of expressive language skills to age (0.002, P < 0.01), schistosomiasis i.e. vocabulary* 1* (0.024, P < 0.05) and narrative skills (0.001, P < 0.01) and soil-transmitted helminths i.e. vocabulary* 1* (0.006, P < 0.05), colours (0.029, P < 0.05) and narrative skills (0.001, P < 0.01) in phase 2 with small to high Cohen’s *d* effect size for various language subtests.

**Conclusion:**

We concluded that even mild schistosomiasis and STH may compromise the performance of preschool children on expressive language. However poor ability in following instructions may have contributed to general poor performance across the two groups tested. Diet, school effect and stunting did not influence the performance of the children on expressive language.

**Supplementary Information:**

The online version contains supplementary material available at 10.1186/s12879-022-07260-2.

## Background

Schistosomiasis, also known as Bilharzia, is a chronic debilitating disease, whose public health importance is second to Malaria, as the most common parasitic condition in Africa [1]. Two main species of schistosomes infect human beings in Africa—*Schistosoma haematobium* and *Schistosoma mansoni* with each species determined by presence of a suitable snail host and its distribution. About 4 million people, mostly children, are infected with schistosomiasis in South Africa [1]. Schistosomiasis is transmitted to humans through contact with contaminated water during bathing, swimming, fishing and domestic chores. Thus, the disease is more prevalent in coastal and rural areas of South Africa where there is no running or tap water [2]. Incidentally, the prevalence of schistosomiasis overlaps with that of soil-transmitted helminths [2]. The prevalence of soil-transmitted helminths (STH) in KwaZulu-Natal is estimated at 38.8% in school aged children [3].

Parasitic worms affect human nutrition and growth through a number of routes depending on the species, the mixture of species, the duration of infection and number of worms [4]. For instance, intestinal worms feed on the contents of the host’s gut and tissues including blood and serum resulting in loss of iron and protein. They can cause maldigestion or malabsorption of the nutrients or induce inflammatory responses that may affect appetite and food intake thereby modifying metabolism and storage of key nutrients. Additionally, contingent responses to infection such as fever and hypertrophy of the muscles also affect behaviour, concentration levels and changes anthropometric status [4]. The effect of schistosomiasis and STH on cognitive function could occur as a result of one or a combination of symptoms associated with parasitic infection including iron deficiency anaemia. Since early childhood is a period of rapid growth and development of all organ sytems, particularly the brain, early life iron deficiency results in abnormal neuronal structure. Hence, schistosomiasis has been associated with negative effects on school attendance, scholastic achievement, learning and memory [5].

Studies investigating neurological or developmental effects of STH infections [6], malaria and anaemia [7] have reported delayed motor and speech development. Neurological consequences are reported even in non-anaemic deficiencies where the potential for learning is reduced due to poor recognition memory and executive functions of infected learners [7]. Early treatment of children with parasitic infections could potentially mitigate the memory and learning deficits in preschool children [5]. There is also empirical evidence indicating cognitive and educational benefits of STH treatment [8].

Since infection with STH and schistosomiasis are confounding factors to cognitive impairment and developmental delays in communities with poor water and sanitation, there is a knowledge gap on whether STH and schistosomiasis have the same effect on expressive language skills as much as they may have on other cognitive skills. Our study aimed at determining the effect of schistosomiasis and STH on expressive language skills among isiZulu speaking pre-schoolers. We determined the profile of expressive language skills of isiZulu speaking preschool children in terms of age, gender, school, stunting and parasitic infection; and compared expressive language skills for children with and without schistosomiasis and STH.

The knowledge we have on isiZulu grammatical pattern is limited, achronological and has not yet produced developmental language norms for receptive and expressive language [9]. We know that isiZulu, like Turkish and Finnish, has characteristically agglutinating morphology and, in many ways, isiZulu is different from English [10]. Data collected from similar Nguni languages such as isiXhosa showed that trilingual and monolingual children had similar lexical development in the languages the children were exposed to [11]. Expressive language builds up from nouns to verbs, adjectives, adverbs, prepositions, conjunctions and auxiliary verbs [12]. Typical emergent skills for expressive language are memory, time-based relations, cause and effect reasoning, social script, sense of self and vocabulary which lead to learning colours, questions and narrative skills can all be achieved as early as 36 months [13].

Expressive language development is usually described using the standards set in English according to the Brown’s stages of syntactic and morphological development where children begin to express their thoughts in single words around 12 months and achieve full sentences and narrative skills by 5 years [14]. Early identification of ‘at-risk children’ usually occurs around the age of 5 years when they are enrolled in a preschool since the provision of appropriate interventions and support can substantially affect future scholastic progress [15]. Since attendance of grade R has been found to influence literacy development, we decided to determine expressive language skills in children attending a preschool [16].

The main risks associated with expressive language development in English speaking children has been shown to include a variety of factors such as family dynamics, interaction with parents, immediate social environment, organic hazards such as brain injury, persistent otitis media, types of food [17], a reactive temperament and being male [18]. Hence, we examined the effect of age, gender, school and status of infection with schistosomiasis and STH on expressive language skills in our sample.

## Methods

### Design and setting of the study

Our study area was a small rural town of Ingwavuma, located in northern KwaZulu-Natal province of South Africa, close to the borders of eSwatini (in the north) and Mozambique (in the east). Ingwavuma is under traditional authority and is regarded as the worst poverty-stricken area in KwaZulu-Natal with an estimated annual income of R32 812 ([19] p. 17). Most people in this area depend heavily on social grants estimated to R13 090 per annum (less than $1000) as their main source of income [19]. The prevalence of *Schistosoma haematobium* among school aged children (over 10 years) in Ingwavuma is 37.5%. However, among the 1–5 years age group prevalence for both *S. mansoni* and *S. haematobium* is 2% [20]. The risk factors for schistosomiasis among young children include the caregiver’s age, type of household head, poor sanitation, access to water source and knowledge about schistosomiasis [21].

The study was an analytical cohort study which described and compared language skills of non-infected and infected children with schistosomiasis and STH using clinical assessments and observations to obtain data. It was an ancillary study to the *Tackling infections to benefit Africa* (TIBA SA) project (http://tiba-partnership.org/about/what-is-tiba). Over 700 preschool children were tested for STH and schistosomiasis in the TIBA study between 2017 and 2020. *Schistosoma haematobium* was diagnosed using the filtration technique on urine samples [22]; *S. mansoni* and STH (*Taenia*, *Ascaris Lumbricoides*, *Trichuris Trichiura)* were diagnosed from stool samples using the Kato Katz technique [23].

Purposeful random sampling was used. To be eligible for the study a child had to be of age between 4.0 and 6.11 years; attend an isiZulu medium preschool or ECD in the target area, have no developmental delays, be monolingual isiZulu speaker and pass a hearing screening test. The study participants were striated based on age (4–6 years), gender (50% of each gender) and inclusion of infection positive participants at a ratio of 2 negative cases for every 1 positive case. The principal researcher was blinded to both status and nature of infection of the study participants. Children were tested in two phases; phase 1 was testing immediately after parasitology screening and phase 2 was testing at least 12 weeks later and after treatment of children with schistosomiasis and STH with oral Praziquantel. Some children participated in both phases while some participated only in phase1 and could not be traced for repeat testing. The data for the children who participated in both phases was treated separately because the phases were 3 months apart (affecting the children’s level of maturity) and the language test was adapted for phase 2 to reduce content bias.

### Sample characteristics

The distribution of children in phase 1 and 2 varied in terms of age, gender and infectious agents as shown in Table 1. The mean age was 4 years 9 months in phase 1 and 5 years 9 months in phase 2. The total number of children positive for schistosomiasis (23%) was less than the number of children positive for STH (33%) with the infectious agents not necessarily co-existing. The spectrum of infections in phases 1 and 2 indicated that STH infection resulted mainly from *A. Lumbricoides* (16%).

**Table 1:** Sample characteristics

Variable	Description	Phase 1	SD	Phase 2	SD
Sample size	Total N	N = 58		N = 48	
Gender	Boys Girls	N = 38 N = 20	0.479	N = 23 N = 25	0.505
Age	Minimum Maximum Mean	4.0 6.11 4.9 years	0.807	4.10 7.03 5.7 years	0.748
Schistosomiasis	Positive (1)	0 = 52 1 = 6 (10.3%)	0.307	0 = 29 1 = 19 (39.6%)	0.392
STH and number of infectious agents in 1 host	STH total 1 agent 2 agents 3 agents	N = 19 (32.75%) 1 = 15.5% 2 = 12.1% 3 = 5.2%	0.590	N = 16 (33%) 1 = (14.5%) 2 = (16.6) 3 = (2.1%)	0.901
Time taken for language test	Mean TTT	26.97 min	12.09	23.71 min	6.49

Seventeen preschools participated in the study; all had pit latrine toilets, one had a rainwater harvest tank within the facility and all of them provided one free meal a day for the children. During our test dates, we observed that the school meals did not include meat and vegetables other than beans. All preschool teachers involved possessed the minimum ECD qualification of high school education and a teaching diploma for Grade R. All 17 preschools had government supplied grade R books, but none had a computer, a television or internet access and generally they all did not have adequate educational resources such as puzzles and board games.

### Data collection procedures

Considering the socio-economic profile of the study area, children were provided with a peanut butter sandwich and orange juice before undergoing testing to ensure they had a healthy breakfast and had energy to participate in the tests. Testing started with hearing screening which, included otoscopic examination and tympanometry to exclude ear infections and its contribution to poor language scores [18]. A nutritionist calculated the body mass index-for-age (weight in kg) and height-for-age (height and arm circumference in cm) to determine stunting classified as mild (1), moderate (2) or severe (3) [24]. The prevalence of stunting was 26%, showing that the majority of participants had adequate nutrition and, no correlation was found between the test scores and stunting in both phases of the study.

All children were monolingual, speaking isiZulu at home and at school. The Developmental Language Test [25], a non-standardised test developed for a research project, was adapted for this study following a pilot study and observations by research assistants. From these observations the test’s vocabulary was adjusted to include local dialect (Northern KwaZulu-Natal coast) and the sequence of test items was formatted into 5 sections for easy scoring and interpretation as shown in Table 2. New test illustrations were developed using pictures taken in Ingwavuma with local community members for improved clarity and to accommodate the adjusted format of the test (see Additional file 1: Appendix A for test illustrations and B for test form).

**Table 2:** Adapted developmental language test score form phase 1 and 2 [25]

Subtest	Task	Changes in phase 2	Sscore based on detail				
			4	3	2	1	0
1. Vocabulary 1 (nouns)	Sing a song						
	Identify people and names	Names					
2. Colours	Recognition	Sequence of colours					
	Naming						
3. Answering WH-questions	Who						
	What						
	Where						
	Why						
4. Vocabulary (verbs and object function)	Counting1–15						
	Object naming	Type of objects					
	Object function						
	Meanings						
	Categorisation						
5. Narrative skills	Inferencing						
	Story retelling	Characters in illustrations					
Total (Sum)	Whole test						

The adapted Developmental Language Test showed a sensitivity of 89.7% in phase 1 and 81.3% in phase 2 reflecting that children who tested positively were true positives while specificity was 10.3 and 18.8%, respectively. The Cronbach’s Alpha was determined to be 0.869 (SD = 5.1) in phase 1 and 0.813 (SD = 7.7) in phase 2 demonstrating adequate internal consistency and suggesting that all items measured the same construct.

The test was administered to one child at a time by the principal researcher who is a speech- language therapist and an isiZulu speaker, familiar with dialect and culture of the area. Scoring points varied from 0 to 4 points per target, depending on the extent of the answer as described in the test form (Additional file 1: Appendix B). Scoring was immediate and automated via the kobo collect app, an open source platform used for collecting and analysing data [26].

### Data analysis

Quantitative data analysis for both phases comprised of descriptive frequency analysis, Independent paired samples t-tests, ANOVA and Bivariate correlations on the SPSS (Statistical package for Social Scientists, v. 25, IBM, Chicago, IL, USA). Post hoc tests were conducted using Bonferroni corrections to measure the specific contribution of variables such as age, gender, school and infection on language categories and time taken to complete the test. The overall error rate was controlled by the use of adjusted significance levels (α = 0.05). Information processing model for cognitive skills [27] and Vygotsky’s sociocultural theory for development and learning guided data analysis and interpretation of scores [28].

## Results

The results showed no significant differences in language scores due to STH or schistosomiasis infections in the pre-treatment Phase 1. However, in Phase 2, there was correlation of schistosomiasis to two language subtests i.e. vocabulary 1 (*r* = 0.024, P < 0.05) and narrative skills (*r* = 0.001, P < 0.01).There was moderate practical significance of schistosomiasis on vocabulary 1 (d = − 0.67), a moderate effect size for colours (d = − 0.53) and a high practical significance for correlation of schistosomiasis to narrative skills subtest (d = − 0.99). The correlation was negative for all three subtests indicating that schistosomiasis contributed to the reduction of the mean. The reader is referred to the extended table of effect size analysis provided in the Additional file 1: Appendix C.

Table 3 shows correlation of variables in phase 2, also indicating correlation of expressive language scores to STH for three subtests i.e*. *vocabulary 1 (*r* = 0.006, P < 0.05), colours (*r* = 0.029, P < 0.05) and narrative skills (r = 0.001, P < 0.01). The correlation effect size of STH on vocabulary 1 was negative (*d* = − 1.84), while for vocabulary 2 it was small and positive (*d* = 0.28). The effect size for colours (*d* = − 1.53) and narratives (*d* = − 2.16) was negative. Statistically, there was no correlation between the test scores and the school factor.

**Table 3:** A comparison of correlation of variables with language test scores in phases 1 and 2

Variables	Phase 1 Language subtests correlations	Phase 1		Phase 2 Language subtests correlations	Phase 2	
		***r***	Sig 2 tailed		**r **	Sig 2 tailed
1. Age	Vocab1 Vocab 2 Colours Narratives	0.338** 0.576** 0.521** 0.473**	0.010 0.000 0.000 0.000	Vocab 1 Colours WH-q	0.390** 0.299** 0.312*	0.006 0.039 0.031
2. Gender	Vocab 1	− 0.300*	0.022*	Vocab 2	0.327*	0.023
3. Schisto + status	sub test 1–5	0.927	NC	Vocab 1 Narratives	0.326* 0.482**	0.024 0.001
4. STH + status	WH-q	0.049	0.260*	Vocab 1 Colours Narratives	0.391** 0.316* 0.454**	0.006 0.029 0.001
5. TTT	Age	− 0.392**	0.002	Age	− 0.316*	0.028

*P < 0.05) **P < 0.01

As indicated in Table 3, There were significant differences in performance by age for four subtests in phase 1 (excluding WH-Questions) and three subtests in phase 2 (excluding narrative skills and vocabulary 2) were established (r = 0.028; P < 0.05). Cohen’s d analysis indicated that the effect was mainly negative, with a moderate practical significance for vocabulary 1 (*d* = -0.84); and a small practical significance for vocabulary 2 (*d* = − 0.21).

Boys performed better than girls for vocabulary subtests 1 and 2, respectively (r = 0.022, P < 0.05) in phase 1 and in phase 2 (r = 0.023, P < 0.05). Similar observations were made for the colours, WH-questions and narratives tests. The correlation effect size for gender was below minimal (*d* = 0.024). The mean time taken to complete the test (TTT) was 26.97 min in phase 1 and 23.71 min in phase 2 and positively correlated to age but not gender (r = 0.002, P < 0.01) for phase 1 and phase 2 (r = 0.28, P < 0.05).

Analysis of the total cohort performance on each subtest is shown in Table 4 and indicate the following trends which are relevant for clinical language norms: In the Vocabulary 1 subtest which, included tasks to recall character names, pointing to characters and objects on picture A, few children achieved above the mean (total score was 12) on the task. For the second subtest which required identification and naming of colours, the majority of children did not know all their primary colour names and achieved the mean score of 3 in both phases. The third subtest required answering interrogative questions in isiZulu where the majority of the children could not obtain the full score in this section mainly because they could not answer the why question. The Vocabulary 2 subtest various aspects of vocabulary including verbs, numbers and categorisation of items. As shown in Table 4, there was minimal variability in the children’s performance.

**Table 4:** Expressive language profile comparison between phase 1 and 2

Subtest	Phase 1 N = 48		Phase 2 N = 58		Sum 1 and 2 Sig	Sig (2-tailed)	
	Mean	SD	Mean	SD	P	Phase 1	Phase 2
Vocabulary1	8.2	2.21	9.0	2.23	0.093	0.098	0.105
Colours	3.1	1.8	3.9	1.3	0.002	0.012	0.009
Wh-questions	2.4	0.84	3.5	0.89	0.539	0.000	0.000
Vocabulary 2	13.1	3.2	20.3	2.3	0.015	0.000	0.000
Narratives	8.2	2.5	6.9	2.2	0.411	0.005	0.004
Sum test 1–5	35.2	8.3	43.8	7.7	0.349	0.000	0.000

Sum 1 and 2 Sig: Sum phase 1 and 2

Overall, the language scores showed similar trends in both phases as there was no significant difference between the means of different language subtests in both phases for the cohorts as depicted in Table 5. The age specific results indicate that age contributed to variation of the total scores with the older children performing better in language subtests.

**Table 5:** Mean raw score per subtest and age band

Age band	N	Mean Voc 1	Mean colours	Mean Q&A	Mean Voc 2	Mean narratives	Mean total
4.0–4.5	8	8	1.8	2.6	12	7.5	32
4.6–4.11	6	7.6	1.5	2.3	12.5	6.6	30.2
5.0–5.5	20	8.25	3.25	2.4	12.45	8.7	34
5.6–5.11	12	9.25	3.17	2.25	13.3	8.4	36.3
6.0–6.5	4	9.75	5	3	16.7	10.5	44.7
6.6–6.11	7	9.4	4.7	3	16.3	11	43.3

Comparing the five subtest’s medians to the 25th and the 75th percentiles, the medians were equidistant from the quartiles indicating normal distribution of data. As shown in Table 6, there was minimal variability in the children’s performance considering the percentile ranks and narrow interquartile range.

**Table 6:** Percentiles

Subtest	25th P	50th P	75th P
Vocab 1	47	51	52
Colours	43	46	51
Wh questions	45	48.5	51
Vocab 2	49	52	52
Narratives	39.5	45	46

## Discussion

The children came from similar backgrounds and comparable schooling contexts hence, no significant differences were noted between the schools and all differentiating variables analysed. The comparison of language scores from the pre-treatment and post treatment phases indicate that the means were higher in phase two. Since we had controlled for content bias by adapting the language test and changing the test pictures in phase 2, the results imply that the older the children the higher the scores in all test areas. The gender comparison in this study shows that boys performed better in vocabulary subtests. This is an unusual finding as many studies report girls as having stronger expressive or oral language skills and boys at a higher risk of speech and language impairment [18, 29, 30]. Since gender related differences in language acquisition have been attributed to cortical sex differences, thought to be acquired or enlarged through different stages of development and attributed to different cognitive strategies between sexes, we recommend further investigation into the factors that may have contributed to the trends in our study, considering its rural setting and specific to isiZulu language.

There was a positive effect of age on TTT, implying that with the increasing children’s age, the speed to complete the test also increased. We regard the effect size minimal as it fell 1 point below the recommended 0.25 [31]. This was an expected finding and in line with the information processing model and cognitive theories of language development such as Luria’s neurological learning theory [32]. We did not find significant correlation between children with and without schistosomiasis and STH in TTT. Uniform processing time was observed for candidates with and without schistosomiasis in learning and memory tests in similar studies [5]. Similarly, when STH was associated with learning, memory and intelligence, there was no significant correlation of testing time with infection [8].

The results showed a negative effect of infection to language subtests where the correlation was significant in the post treatment phase 2 suggesting that children with history of parasitic infections performed lower than their peers; thus, explaining the reduction of the mean of the relevant tests. This negative effect size was medium for vocabulary and colours subtests but high for narratives skills. Narrative tasks required the ability to follow commands and retell a story in sequence, skills that require application of short term (ability to retain information for a short time) and working memory (the ability to maintain activated knowledge in the focus of attention) [33]. There is evidence of a relationship between linguistic knowledge and verbal working memory (vWM) in that linguistic knowledge impact on vWM [34] and that vWM depends on long term representations and processes that are involved in speech production [35]. Therefore, we are intrigued by our results which imply a causal relationship between schistosomiasis and weaker narrative skills in the light of a known relationship between parasitic infection and memory deficits. Furthermore, the findings reveal the possibility of long term deficits in executive functions and memory after the parasitic worm infection and iron deficiencies have resolved [36].

The prevalence of STH and Schistosomiasis in our study was similar to that of other studies conducted in KwaZulu-Natal but less than the prevalence in other low-and middle-income countries [37]. The intensity of infection with both STH and schistosomiasis was mild and the majority of the participants were found to have adequate nourishment. We regard this as an important finding as the participants in this study area were from a typical low socio-economic rural context and their school diet was not particularly rich in recommended nutrients such as omega 3 polyunsaturated fatty acids [38]. Further investigation of the children’s home nutrition and other possible contributing contextual factors would have provided more conclusive results. Thus, one may conclude that the consequences of moderate to severe infection with Schistosomiasis and STH such as malnutrition do not apply to this study population [39]. This finding has positive implications for the accuracy of the profile of language skills reflected by the findings. We recommend involvement of parents through a direct questionnare and to obtain more data on the socio-economic factors impacting on the children’s health related quality of life and specific information on language acquisition.

## Conclusion

Schistosomiasis mainly negatively impacted on language scores, with a moderate practical significance for vocabulary and colours and a high effect for the narrative subtest. Our findings illustrate the contribution of verbal working memory on expressive language abilities. Our findings also showed correlations of expressive language performance to age as well as correlations of vocabulary subtests to gender (boys), STH and schistosomiasis. Socio-economic factors such as diet, preschool attended and stunting did not contribute to the scores. The adapted Developmental Language Test offered valuable information towards our understanding of the profile of abilities of isiZulu speaking preschool children. The findings of this study are relevant for all professionals involved in the education and rehabilitation of preschool children with infectious diseases and emphasise the need for treatment of mild STH and Schistosomiasis in Africa.

## Supplementary Information

**Additional file 1.** Appendix C/ MOEMS 1.

## Data Availability

Data sets used and analysed during the current study are available from the corresponding author on reasonable request.

## Abbreviations

STH: Soil-transmitted helminths
TIBA SA: Tackling infections to benefit Africa–South Africa
ECD: Early childhood development
SD: Standard deviation
ANOVA: Analysis of variance
TTT: Time taken to test
vWM: Verbal working memory
NIHR: National Institute for Health Research
