# Supplementary material for: The effect of schistosomiasis and soil-transmitted helminths on expressive language skills among African preschool children
Source: BMC Infect Dis. 2022 Mar 18;22:264. doi: 10.1186/s12879-022-07260-2 (PMC8931967; doi:10.1186/s12879-022-07260-2)
Supplement: Supplementary file 1 — Additional file 1. Appendix C/ MOEMS 1. [file 12879_2022_7260_MOESM1_ESM.docx]

APPENDICES EXPRESSIVE LANGUAGE TEST

**Appendix A :Adapted Developmental Language Test Illustrations PHASE 2**

**Picture A Picture B**


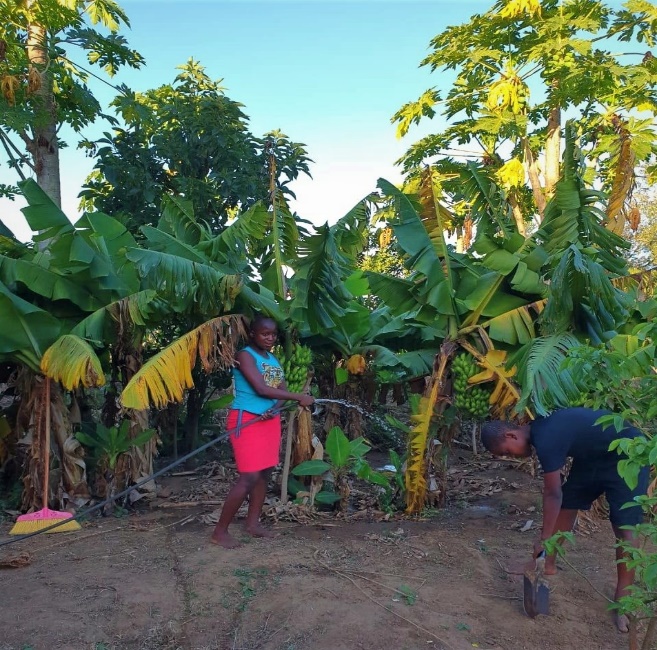

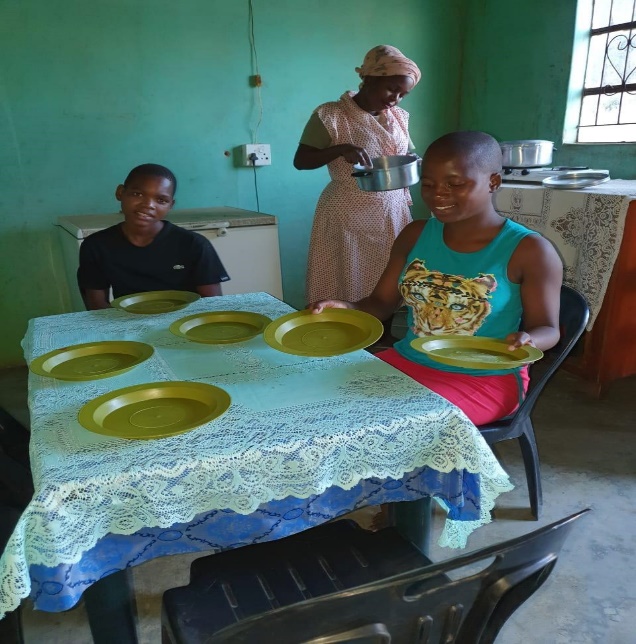


**Picture C Picture D**


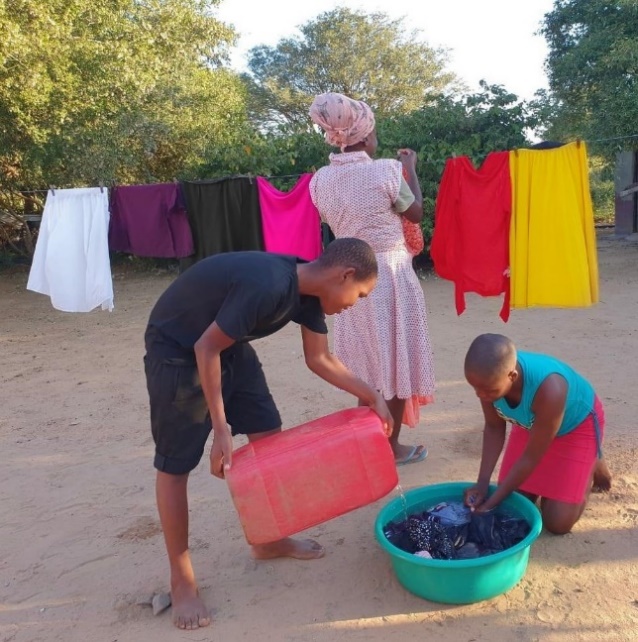

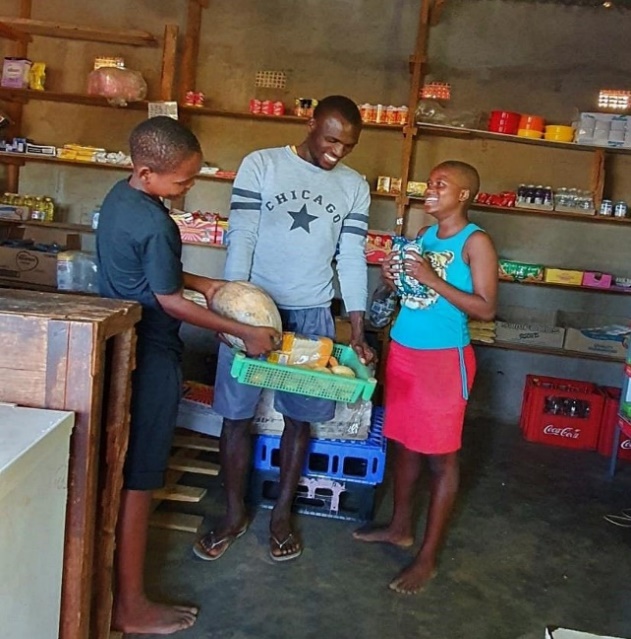


**APPENDIX B Developmental Language test (Adapted for Ingwavuma)**

**SECTION A**

Purpose: Establish rapport &introduce the narrative

1. **Instruction:**

Unalo iculo olithandayo esingalicula ndawonye noma umlolozelo esingawusho sobabili?

| **1**  Song | Song or rhyme production. | **2** | ***1*** | ***0*** |
| --- | --- | --- | --- | --- |
|  |  | *song or rhyme clear with enjoyment* | *sings but lacks clarity* | *unable to perform* |

1. ***Instruction:***

***Show all pictures to the child then proceed with questions:*** Akubuke lezizithombe. Lona umndeni wakwa Mthembu. Kukhona ubaba Kanye nomama, kuthiwa umama nonkosikazi Mthembu nomfana obizwa ngokuthi uThemba nentombazane ebizwa ngokuthi uNosipho.

| 2. Names/ story characters | Ungasho ukuthi bangobani labantu?  nokuthi bangobani amagama abo? | *3* | *2* | *1* | *0* |
| --- | --- | --- | --- | --- | --- |
|  |  | *All characters & their names correct* | *all people correct, some names correct* | *some people, and some names correct* | *No response* |

**SECTION B**

**Picture 1:**

Description:

Izithombe zisitshela indaba yokuthi umndeni wenzani undawonye ngomunye uMgqibelo ekuseni. Buka isithombe sokuqala. Unkosikazi Mthembu washeshe wavuka ukuywasha izingubo. UThemba noNosipho bayamsiza umama wabo.

| 3.1  *Follows 2- and 3- part commands* | Ngikhombisa umama Mthembu, bese ungikhombisa uNosipho, ugcine ngokungikhombisa uThemba. | *3* | *2* | *1* | *0* |
| --- | --- | --- | --- | --- | --- |
|  |  | *all correct* | *all shown, but order incorrect* | *Two people shown in correct order* |  |

| 3.2  WH Q | Bakuphi? (where) | *1* | *0* |
| --- | --- | --- | --- |
|  | Benzani? (what) | 1 | 0 |
|  | Bakwenzelani lokho? (why) | 1 | 0 |
|  | Ubani owasha/ oshuka izingubo? (who) | 1 | 0 |
|  | Uzineka kuphi unkosikazi Ndaba izingubo? (where) | 1 | 0 |
|  | Ukwenzelani lokho? (why) | 1 | 0 |

| 3.3  *Recognizes*  *colors* | Ungangikhombisa into emnyama (black) esithombeni? | *1* | *0* |
| --- | --- | --- | --- |
|  | Ungangikhombisa into e orange? | *1* | *0* |
|  | Ungangikhombisa into emhlophe (white) | *1* | *0* |
|  | Bumbala muni utshani? | *1* | *0* |
|  | Uwumbala onjani lamaplate? | *1* | *0* |

Ngizobala amaphekisi. Ngisize ukuwabala?

(count to 5 and wait for the child to copy this. If s/he can do this continue counting to 10 and wait for the child. If s/he can do this, count the remaining pegs).

| 3.4 Counting 1-15 | C*ounting to 15.* | *3* | *2* | *1* | *0* |
| --- | --- | --- | --- | --- | --- |
|  |  | *counts to 15* | *counts to 10* | *counts to 5* | *less than 5/ NR* |

**SECTION C**

# Picture 2

Sebeqedile ngokuwasha izingubo oThemba no Nosipho bayakosiza umnumzane Ndaba ngesikhathi esebenza etshala izimbali engadini

| 4.1 Identify object by name | Ngikhombisa ifosholo | | 1 | 0 |
| --- | --- | --- | --- | --- |
|  | Yini enye onganisela ngayo engadini? | | 1 | 0 |
|  | Ngikhombisa umshanelo | | 1 | 0 |
|  | Ngikhombisa amahlamvu/ amacembe | | 1 | 0 |
| 4.2 Understand object function | Ubambeni/uphetheni uNosipho? | | 1 | 0 |
|  | Eyokwenzani? | | 1 | 0 |
|  | uyasiza yini umshanelo ensimini? | | 1 | 0 |
|  | Yini enye onganisela ngayo engadini? | | 1 | 0 |
|  | Elokwenzani ifosholo? | | 1 | 0 |
| 4.3 Understands differences in meaning OPPOSITES |  | Ubani omile ubani obheke phansi? | 1 | 0 |
|  |  | ungikhombise isibhakabhaka. | 1 | 0 |
|  |  | Ngikhombise isihlahla esifishane. | 1 | 0 |
|  |  | Ngikhombise isihlahla eside. | 1 | 0 |
|  |  | Sinaziphi isithelo isihlahla esiseduze kuka Nosipho. | 1 | 0 |
|  |  | Ngikhombise inhlabathi. | 1 | 0 |

# SECTION D

# Picture 3

Okulandelayo umnumzane Mthembu baya kothenga noThemba Kanye noNosipho

| 5.1  Fruit & vegetables *Naming* | Can you tell me all the names of fruit you know? | *3* | 2 | 1 | 0 |
| --- | --- | --- | --- | --- | --- |
|  |  | *more than 4 correct* | *more than 2 correct* | *1 correct* | *None offered* |
| 5.2  Fruit/ Vegetable *Differentiation* | Nhloboni yokudla abakuthengile (prompt: ingabe amafruits noma ama vege?) Yimaphi amanye ama veji owaziyo noma akhona ensimini ekhaya? | 3 | 2 | 1 | 0 |
|  |  | *more than 4 correct* | *more than 2 correct* | *1 correct* | *None offered* |
| 5.3  Narratives | Uke waya esitolo kothenga?  Ngitshele/ngixoxele ngako.  *(Prompts if necessary: Uye kuphi? Waya nobani? Nafika kanjani esetolo? Nathengani?* | *3* | 2 | 1 | 0 |
|  |  | *Unprompted, 3 details* | *Prompted 3 details* | *Prompted less than 3 details* | *No response/ cannot relate* |

**SECTION E**

# Picture 4

Ekugcineni baqeda ukuthenga. Uma befika ekhaya uThemba no Nosipho bathola ukuthi umama wabo esebalungisele isidlo sabo sasemini base bemsiza ukudeka itafula.

| 6.1. Counting OBJECTS | Zingakhi izitsha ozibonayo etafuleni? | **1** | | | | **0** | | | |
| --- | --- | --- | --- | --- | --- | --- | --- | --- | --- |
|  |  | Correct | | | | Incorrect | | | |
| 6.2. Associating  Colours | Awumbala onjani amapuleti futhi kukhona yini omunye enye into ewumbala ofana nawo? | 2 | | 1 | | | | 0 | |
|  |  | Both answers correct | | 1 answer correct | | | | Both answers wrong | |
| 6.3.  Inferencing | Ucabanga ukuthi lomndeni uzokwenzani manje? | 2 | | 1 | | | | 0 | |
|  |  | Answer makes sense, future tense | | Answer correct tense incorrect | | | | Answer incorrect | |
| 6.4.  Narratives | Accurately relays a whole story Ungangixoxela indaba yonke ngabakwenzile abakwa Mthembu ngomgqibelo ekuseni? | **4** | 3 | | 2 | | 1 | | 0 |
|  |  | 4 events | 3 events | | 2 events | | 1 event | | Could not tell the story |

**APPENDIX C: PHASE 2 Size Of Effect Analysis Of Infection On Language Tests**

| **Measurement description**  **t -tests** | **Cohen’s d**  **( d=m1-m2/sd)** | **Interpretation**  **(.8=large;**  **.5 moderate; .2 small)** |
| --- | --- | --- |
| **SCHISTOSOMIASIS POSITIVE** | | |
| Z score (sum vocab 1) & Schistosomiasis | d= -.671 | Medium Negative effect |
| Z score (Sum Vocabulary 2) & Schistosomiasis | d= -.188 | Minimal significance |
| Z SCORE (sum colours) & Schistosomiasis | d= -.532 | Medium Negative effect |
| Z score (sum WH- questions) & schistosomiasis | d= -.255 | Small negative effect |
| Z score (Sum Narratives) & Schisto | d= -.99 | High negative effect |
| **SOIL TRANSMITTED HELMINTHS POSITIVE** | | |
| Z score ( Sum Vocab 1) & STH | d= -1.84 | Minimal significance |
| Z score (Sum Vocab 2) & STH | d=.28 | Small effect |
| Z score (Sum WH questions ) & STH | d= -.62 | Minimal significance |
| Z score (Colours) & STH | d= -1.41 | No effect |
| Z score (Sum Narratives) & STH | d= -2.16 | Minimal significance |

***P<0.05)**
